# Effect of Gender and Age on the Correlation between Helicobacter pylori and Colorectal Adenomatous Polyps in a Chinese Urban Population: A Single Center Study

**DOI:** 10.1155/2020/8596038

**Published:** 2020-02-10

**Authors:** Xia-Xia Zhao, Ming-Hao Liu, Rui-Ling Wang, Tian Tian

**Affiliations:** ^1^Postgraduate Training Base of Jinzhou Medical University, The PLA Rocket Force Characteristic Medical Center, Digestive Internal Medicine, Beijing, China; ^2^The PLA Rocket Force Characteristic Medical Center, Digestive Internal Medicine, Beijing, China

## Abstract

**Objectives:**

To investigate whether Helicobacter pylori (H. pylori) infection increases the risk of colorectal adenomatous polyp (CAP) in the context of age and gender.

**Methods:**

A total of 563 study subjects (male/female, 368/195) from Beijing, China, with higher nursing level who underwent colonoscopy were retrospectively collected. H. pylori and CAP were detected by carbon-13 urea breath test and colorectal colonoscopy. The correlations between the number, size, distribution, and pathological grade of CAP and H. pylori infection were analyzed. The population was further stratified by age and gender in order to examine the risk of H. pylori and CAP in the context of these variables. The influence of H. pylori on the risk of CAP was assessed by logistic regression model.

**Results:**

315 participants were diagnosed with CAP, and 207 participants were classified as healthy controls. The prevalence of H. pylori in the CAP group was significantly higher than that in the healthy control group (119/315, 37.8% versus 44/207, 21.3%) (*p* < 0.001). The proportion of H. pylori positive plus CAP in participants <50 years old was significantly higher than that in participants >50 years old (87/250; 34.8% versus 32/65; 49.2%) (*p* < 0.001). The proportion of H. pylori positive plus CAP in participants <50 years old was significantly higher than that in participants >50 years old (87/250; 34.8% versus 32/65; 49.2%) (*p* < 0.001). The proportion of H. pylori positive plus CAP in participants <50 years old was significantly higher than that in participants >50 years old (87/250; 34.8% versus 32/65; 49.2%) (*p* < 0.001). The proportion of H. pylori positive plus CAP in participants <50 years old was significantly higher than that in participants >50 years old (87/250; 34.8% versus 32/65; 49.2%) (

**Conclusions:**

H. pylori is a major risk factor for CAP. Further studies are needed to assess the effects of H. pylori treatment or persistent infection on the occurrence or recurrence of CAP.

## 1. Introduction

Colorectal cancer (CAC) is one of the common malignant tumors of the digestive system in China. The incidence rate of CAC ranks third and its fatality rate ranks fourth worldwide [1]. While incidence of CAC is high in Europe and the United States, it is low in Africa and central Asia. Among Western populations, the risk of terminal CAC is at about 5% [2]. Therefore, colorectal adenomatous polyp (CAP) has been regarded as the most critical precancerous disease, and its identification and treatment are crucial for cancer prevention. Since early detection of colorectal adenomas can prevent the development of CAC, identification of patients who are at high risk for colorectal adenomas and subsequent surveillance for signs of CAP is extremely important. Although certain risk factors, such as family tumor history and familial adenomatous polyposis, could contribute to CAC, 85% of CAC cases cannot be related to specific risk factors [3].

CAPs are precancerous lesions that usually develop into malignant tumors by the “adenomatous-atypical hyperplasia-carcinoma” sequence, and 75% of CAC can be traced back to this process [4]. Generally, CAP takes at least 10 years to progress into malignant tumors [5]. However, only when doctors and patients prioritize CAP screening and treatment can malignant transformation be prevented. Risk factors that can be used for screening CAP, including smoking, obesity, alcohol, NSAID, and dietary factors, are associated with the development of CAP and CAC [6].

H. pylori infection rate is as high as 50% worldwide [7]. About half of the population in China, Iran, and other developing countries are infected with H. pylori [8]. Around 4.4 billion individuals were diagnosed as positive with H. pylori worldwide [9, 10]. Accumulating evidence has indicated that H. pylori infection is an important risk factor for gastric ulcer, gastritis, malignant tumor, and other systemic diseases. Previous study also suggested that H. pylori might be associated with a risk of CAC [6], while a meta-analysis also showed an approximately 20-40% increased risk of CAC in H. pylori [11]. The aim of this study was to evaluate the association between H. pylori infection and colorectal adenomas in a Chinese population and investigate if severity of the adenomas affects the strength of such association.

## 2. Methods and Materials

### 2.1. The Population and Ethical Guidelines

In this study, we determined the participants by considering the clinical information of people who had undergone full-length colonoscopy from March 2016 to June 2019 at the PLA Rocket Force Characteristic Medical Center in China. The factors (see exclusion criteria below) that seriously affect the results of this paper were excluded. Results were not distributed to study participants. This is a retrospective study, and we collected the clinical information of the participants retrospectively without any effect on the participants, and their information were not disclosed. The research program is in line with the ethical guidelines of the 1975 Helsinki Declaration (6th revision, 2008), as reflected in the prior approval of the agency's human research council.

### 2.2. Study Design and Exclusion Criteria

A total of 713 participants (male/female, 458/255) were incorporated into our study; people with similar socioeconomic status and undergone colonoscopy were retrospectively collected. After 150 cases (male/female, 90/60) who did not meet the research requirements were eliminated, 563 people (male/female, 368/195) who met the requirements remained. After a night of fasting, two breath samples were collected every 30 minutes for detection of 13C-UBT with a H. pylori kit (ISO Diagnostic Canada). The samples were analyzed by meteorological chromatography, and the results were expressed as delta over baseline (DOB). To measure the fasting plasma glucose, 15 ml fresh blood was collected from the anterior cubital vein after 12 hours of fasting, and biochemical tests were performed within 4 hours. All those (*n* = 150) who met the following criteria were excluded: (i) participants with poor bowel preparation, incomplete endoscopic examination, or unknown H. pylori infection status (*n* = 30); (ii) participants with inflammatory bowel disease, familial neoplasia, or Gardner's syndrome (*n* = 45); (iii) participants with CAC, severe systemic disease, or a history of gastrointestinal surgery (*n* = 72); and (iv) participants who took bismuth agent in the last four weeks, used antiseptic or proton pump inhibitors for two weeks, or took H2 receptor antagonist (*n* = 13).

### 2.3. Management and Classification of Colorectal Polyps

All subjects had undergone whole colon examination using Olympus CF colonoscopy, and the location, size, shape, and number of polypoid lesions were recorded. According to the size of the largest polyps, patients with colorectal CAP were divided into three groups: 0-10 mm, 10-20 mm, and >20 mm. According to the number of CAP examined by full colonoscopy, single polyp was defined as 1 polyp, and multiple polyps was defined as ≥2 polyps. The observed colorectal CAPs were resected, collected, and classified as either right colon (CAP collected from the transverse colon near the hepatic curvature, ascending colon, and ileocecal region) or left hemicolon (CAP from the transverse colon middle section, colon splenic curvature, descending colon, sigmoid colon, and rectum). All the specimens were paraffin-embedded. Moreover, we categorized the study population into two age groups: <50 years old and ≥50 years old, due to the higher risk of CAC in ≥50-year-old asymptomatic, average-risk patients [12].

### 2.4. Statistical Analysis

Statistical analysis was performed using SPSS software version 22.0 (SPSS Inc., Chicago, IL). The distributions of continuous and categorical variables were analyzed with Student's *t* test and chi-squared test, respectively. A multivariable logistic regression analysis was adopted to estimate the predictive effect of H. pylori on the risk of CAP. *p* < 0.05 was considered statistically significant.

## 3. Results

### 3.1. Characteristics of the Participants

A total of 356 patients were diagnosed, among which 315 (male/female, 190/125) were diagnosed with CAP, 41 (male/female, 20/21) were diagnosed with inflammatory hyperplastic polyp (IHP) by colorectal biopsy, and 207 subjects were classified as healthy controls. The average age was 53.2 ± 7.75 for healthy controls and 57.27 ± 10.4 for CAP subjects, indicating that the CAP group was older than the control group (*p* < 0.001) (Table 1). Both groups predominantly consisted of males, while the ratio of females in the CAP group (125/315, 39.7%) was significantly higher than that in the control group (49/207, 23.7%) (*χ*^2^ = 14.41, *p* < 0.001) (Table 1). Participants with CAP exhibited a significantly higher body mass index (BMI) (24.96 ± 3.65 in CAP versus 23.57 ± 3.96 in control) and blood sugar level (5.66 ± 1.65 in CAP versus 5.16 ± 0.66 in control) than healthy controls (*p* < 0.001 for both BMI and blood sugar). However, no significant differences were observed in cholesterol and triglycerides between the two groups (*p* > 0.05) (Table 1). The portion of women ≥50 years old in the CAP group (111/125, 88.8%) was higher than that in the healthy control group (34/49, 69.4%) (*χ*^2^ = 10.01, *p* = 0.002). However, no statistically significant differences were observed in the portions of males ≥50 years old between the CAP (139/190, 73.2%) and the control (102/158, 64.6%) groups (*p* = 0.083, Table 2).

### 3.2. The Prevalence of H. pylori Infection in CAP

As shown in Table 1, the H. pylori infection rate in the CAP group (119/196, 37.76%) was significantly higher than that in the healthy control group (44/163, 21.26%) (*χ*^2^ = 15.88, *p* < 0.001). As shown in Table 1, in the CAP group, the H. pylori positive ratio in male (83/190, 43.68%) was significantly higher than in female (36/125, 28.8%) (*K* = 0.276, *p* < 0.001). The infection rate of H. pylori in participants aged ≥50 (87/250, 34.8%) was significantly lower than that in those aged <50 (32/65; 49.2%) (*k* = 0.04; *p* = 0.033) (Table 2). Two chi-squared test showed that the H. pylori infection rate in the CAP group (38.49%) was significantly higher than that in the IHP group (34.15%, *p* < 0.001, Table 3). In the CAP group, the H. pylori infection rate in 0-10 mm polyp group (101/173, 58.34%) was significantly higher than that in 10-20 mm (32/73, 43.8%) and >20 mm (8/25, 32%) group (*p* < 0.001, Table 3). With regard to pathological classification, low-grade CAP was the predominant category (270/315, 85.71%; Table 3). Furthermore, a significant difference was observed in H. pylori infection between low-grade (100/270, 37%) and high-grade CAP (19/45, 42.2%) groups (*p* < 0.001; Table 3). Most CAPs were located in the left hemicolon (74.43%), while both the location and number of CAPs did not show any statistically significant association with H. pylori infection (*p* > 0.05). In our study, the CAP positive rate (315/356, 88.5%) was much higher than that of IHP (41/356, 11.5%) (*p* < 0.001).

### 3.3. H. pylori Infection Is Associated with a Higher Odds Ratio of CAP

Multiple logistic regression models were used for analysis of H. pylori in the context of CAP. H. pylori infection demonstrated a higher odds ratio (OR) of CAP (OR = 2.679; 95% CI: 1.717-4.179, *p* < 0.001). Age, gender, BMI, cholesterol, and blood sugar were also found to be risk factors for CAP (*p* < 0.05). While the level of triglycerides was not associated with CAP (*p* > 0.05). After adjusting for age, gender, BMI, cholesterol, and other risk factors, H. pylori infection remained as a risk factor for CAP (OR = 2.477, 95% CI: 1.627-3.77) (Table 4).

## 4. Discussion

It is well known that at least 15% of all incidences of cancers are caused by infection. H. pylori was recognized as a class I carcinogen of gastric cancer, and its important role in the development of gastric cancer, gastric mucosa-associated lymphoid tissue lymphoma, and other diseases has been proven [13]. However, the relationship between H. pylori infection and CAC is still unclear. A previous study has shown that H. pylori infection can upregulate the expression of basal metalloproteinase [14], which may be involved not only in the occurrence of CAC in CAP but also in the invasion and metastasis of CAC [15]. According to other studies, infection by H. pylori may increase the release of gastrin, which acts on intestinal epithelial cells and stimulates the production of cox-2, a well-known player in the occurrence, development, invasion, and metastasis of CAC [16–18]. Meanwhile, serological response to Cag A (cytotoxin-associated gene A) and Vac A (vacuolating cytotoxin A) secreted by H. pylori is associated with a higher risk of CAP and/or CAC [19–21]. More evidence showed that the histopathological severity of CAC is associated with H. pylori infection, and H. pylori infection can increase the risk of developing CAC in people under the age of 50 who smoke [22–24]. However, on the other hand, a few reports have also stated that CAP and CAC are unrelated to H. pylori infection [25, 26]. It has been reported that H. pylori strains are very diverse demographically, while the majority of published studies examining the association between H. pylori and CAC risk did not take into account the heterogeneity of H. pylori [27, 28]. Studies have reported that the heterogeneity of demographic characteristics (race, geographical environment, gender, age, etc.) will affect the research results [21]. In this study, we stratified the participant population based on gender, age, and histopathologic degree of CAP to study the relationship between H. pylori infection and CAP in Chinese people.

CAP is widely known to be an essential process in the development of CAC, and its occurrence is correlated with age, gender, and metabolic abnormalities (BMI, blood glucose, and blood lipids) and hyperinsulinemia [29, 30]. Abnormal metabolism of blood glucose and lipid results in the release of a large amount of factors into the blood including free fatty acids, growth factors, sex hormones, adipokines, and some proinflammatory chemokines; these factors play key roles in regulating dysplasia and malignant transformation [31]. Abnormal metabolism of hyperinsulinemia can directly or indirectly stimulate the growth of colon epithelial cells [32]. H. pylori infection is known to be associated with metabolic syndrome, a complex set of metabolic disorders. This may be due to that chronic inflammatory response induced by H. pylori infection that can upregulate the expression of C-reactive protein (CRP), tumor necrosis factor-alpha (TNF-*α*), and interleukin 6 (IL-6), which can be activated by the signaling pathway of nuclear factor kappa B kinase (NF-*κ*B) [33]. In addition, chronic H. pylori infection may activate a variety of cytokines such as IL-6, interferon gamma, and TNF-*α* through liposomes, thereby inhibiting lipoprotein lipase activity, and decreased lipoprotein lipase activity leads to reduced degradation of very low-density lipoproteins (VLDLs) and increased total cholesterol and triglycerides, ultimately leading to increased blood lipids [34]. Finally, H. pylori infection produces Cag A, which affects glycosylation, and H. pylori infection also leads to insufficient secretion of pepsin, which causes abnormal metabolism of glucose, triglycerides, and uric acid [29, 30]. All these further clarify the correlation between H. pylori and CAP [35, 36]. Our study showed that CAP was significantly correlated with BMI and blood sugar but not triglycerides and cholesterol (Table 1); the possible reason is that our sample size is relatively small and the data have deviation, so it is necessary to increase the sample size in our future study to further address their correlation. In addition, in this study, the prevalence of female adenomatous polyps was higher (125/315, 39.6%) than in the control group (49/207. 23.7%) (*p* < 0.001, Table 1). Estrogen level has been suggested to play a role in promoting the development of CAC and CAP [37]. In addition, a study reported that women may be an independent protective factor for H. pylori infection [38]. In this study, we found that in the CAP group, the H. pylori infection rate in male (83/190, 43.6%) was significantly higher than in female (36/125, 28.8%). The effect of H. pylori infection on the relationship between CAP and sex hormone still requires further investigation. Besides, a higher H. pylori infection rate was correlated with <50 years old in CAP (*p* = 0.033, Table 2), indicating that H. pylori infection is more common in people aged <50, which may be involved in the occurrence and development of CAP; similar conclusions have been found in other literature [24].

Our study showed a much higher H. pylori infection rate in CAP group than that in IHP group and polyp-free control group, consistent with a previous study which showed that the high pathological grade of CAP is consistent with the high infection rate of H. pylori (*p* < 0.001) [30]. In addition, it was reported that the correlation between H. pylori and CAC increased with the increase of the histopathological severity of colon tumors. Similarly, the strength of the association increased with the size and number of adenomas [39]. In this study, H. pylori was correlated with CAP size and type of pathology (*p* < 0.05, Table 3), showing that H. pylori may be involved in the progression of CAP and the pathological grading; however, the number of adenomas was not significantly correlated with H. pylori (*p* > 0.05, Table 3). Several studies have shown that H. pylori infection is associated with proximal CAP [39, 40], while in this study, CAP was mainly distributed in the left colon and was not associated with H. pylori infection (*p* > 0.05, Table 3). This difference may be attributed to the differences in H. pylori detection (serological versus nonserological methods) and specimen location (stomach versus colon) [11, 21] or may be due to differences in host and H. pylori polymorphism or research methods, which needs further study. Our study showed that the OR of H. pylori infection in adenoma patients was 2.679 (95% CI: 1.717-4.719) compared with that in the control group. After adjusting for age, gender, BMI, cholesterol, and other risk factors, H. pylori infection was proved to be a risk factor for CAP (OR = 2.477, 95% CI: 1.627-3.77), which further confirms the relationship between H. pylori and CAP. Another study confirmed the correlation, showing that the mucous membranes of colon cancer had a higher DNA rate of H. pylori than normal mucous membranes [41], confirming that H. pylori is an integral factor in CAP/CAC. It is suggested that active bactericidal treatment is needed for the treatment of CAP patients with H. pylori infection, which may have positive significance in preventing the occurrence of CAC. For the first time, we grouped by gender and age to investigate the correlation between H. pylori and colorectal adenoma polyps. We also considered other demographic or clinical confounding factors (age, gender, BMI, blood sugar, etc.) that could influence the development of CAP. However, due to our patients being from a single gastroenterology group practice in one geographic area in the single-center study, our findings may not be generalizable to patients in other settings and communities.

## 5. Conclusion

This is a study conducted in an area with a high prevalence of H. pylori infection. Our study showed different H. pylori infection rates in different aged people with different genders, and that woman is a risk factor for CAP. Besides age, gender, and BMI, H. pylori infection demonstrated an independent risk factor for CAP and is related to the size and location of CAP. Further studies are needed to assess the effects of H. pylori treatment or persistent infection on the occurrence or recurrence of CAP, and to exclude as many factors affecting CAP as possible, such as lifestyle and the detection technology of H. pylori. Moreover, studies with larger sample size could improve the detection rate of H. pylori and provide a clearer understanding of H. pylori and CAP.

## Figures and Tables

**Table 1:** General characteristics of a population.

Classification	CAP group	Control group	*p* value
Gender (male/female)	190/125	158/49	<0.001^a^
			<0.001^a^
Age	57.27 ± 10.4	53.2 ± 7.75	
Male (≥50/<50)	139/51	102/56	0.083
Female (≥50/<50)	111/14	34/15	0.002
H. pylori infection (positive/negative)	119/196	44/163	<0.001^a^
BMI	24.96 ± 3.65	23.57 ± 3.96	<0.001^a^
Cholesterol	4.56 ± 0.9	4.69 ± 0.85	0.092
Triglycerides	1.6 ± 1.07	1.43 ± 0.97	0.069
Blood sugar	5.66 ± 1.65	5.16 ± 0.66	<0.001^a^

^a^Represents a *p* value less of than 0.05 between the CAP group and control group.

**Table 2:** H. pylori and CAP.

Category	H. pylori positive	H. pylori negative	*p* value
Male	83	107	<0.001^a^
Female	36	89	
Age			
≥50	87	163	0.033
<50	32	33	

^a^Represents a *p* value less of than 0.05 between H. pylori positive and H. pylori negative groups. Male and female (*p* = 0.00).

**Table 3:** CAP distribution/location/quantity/pathological type and H. pylori.

		H. pylori positive	H. pylori negative	*p* value
Location^∗^	Right side of colon	69	98	>0.05
	Left side of colon	79	148	
	The rectum	44	57	
Number	Single	31	66	>0.05
	Multiple	88	130	
Size^∗∗^	0-10 mm	101	72	<0.001^a^
	10-20 mm	32	41	
	>20	8	17	
The pathologic types	CAP	117	187	<0.001a
	IHP	14	27	
	Low-grade CAP	100	170	<0.001a
	High-grade CAP	19	26	

^a^Represents a *p* value less than 0.05 between H. pylori positive and H. pylori negative group. (^∗^left and right semicolon (*p* = 0.9), left and rectum (*p* = 0.130), and right and rectum (*p* = 0.718). ^∗∗^0-10 mm and 10-20 mm (*p* = 0.036), 0-10 mm and >20 mm (*p* = 0.013), and 10-20 mm and >20 mm (*p* = 0.029).

**Table 4:** Multiple logistic regression analysis.

Category			95% CI					Adjusted 95% CI		
	*B*	OR	Upper limit	Lower limit	*p* value	Adjusted *B*	Adjusted OR	Upper limit	Lower limit	*p* value
Age	0.034	1.034	0.013	1.056	0.002^a^	0.043	1.044	1.024	1.064	<0.001^a^
H. pylori	0.985	2.679	1.717	4.719	<0.01^a^	0.907	2.477	1.627	3.77	<0.001^a^
BMI	0.133	1.142	1.067	1.222	<0.01^a^	0.117	1.125	1.162	1.191	<0.001^a^
Gender	-1.071	0.343	0.216	0.544	<0.01^a^	-0.91	0.403	0.259	0.427	<0.001^a^
Blood sugar	0.409	1.505	1.168	1.939	0.002^a^	0.388	1.474	1.153	1.884	0.002^a^
Cholesterol	-0.271	0.763	0.605	0.962	0.022^a^	-0.248	0.78	0.621	0.979	0.032^a^
Triglycerides	0.115	1.122	0.896	1.406	0.315	0.123	1.21	0.978	1.313	0.311

^a^Represents *p* < 0.05.

## Data Availability

The [Retrospective] data used to support the findings of this study are restricted by the [Ethics committee of rocket army characteristic medical center] in order to protect [Patient privacy]. Data are available from [Rui-Ling Wang, Email: wrl931096@163.com] for researchers who meet the criteria for access to confidential data.
